# Review of Lot Quality Assurance Sampling, Methodology and its Application in Public Health

**DOI:** 10.3126/nje.v9i3.24507

**Published:** 2019-09-30

**Authors:** Rama Shankar Rath, Hariom Kumar Solanki

**Affiliations:** 1 Assistant Professor, Department of Community Medicine and Family Medicine, All India Institute of Medical Sciences, Gorakhpur, Uttar Pradesh, India.; 2 Assistant Professor, Department of Community Medicine, Dr. Baba Saheb Ambedkar Medical College, Rohini, Delhi, India.

**Keywords:** Rapid assessment, Epidemiological method, LQAS, Monitoring and Evaluation

## Abstract

Rapid collection of data is of utmost importance in monitoring and evaluation of activities of public health importance. Among others techniques, 30 by 7 cluster sampling and Lot quality assurance sampling(LQAS) methods have been described in literature for this purpose. However, LQAS is often sparingly used in most settings, undermining its importance as a effective epidemiological tool in public health practice. To some extent LQAS is inadequately understood and even less emphasized method, especially in the postgraduate teaching and training. In this paper we aim to explain the use, method and application of LQAS in public health settings as well as discuss common pitfalls to avoid while planning and drawing inferences based on data collected through LQAS.

## Introduction

Sampling strategy is one of the core considerations while planning any epidemiological study. Using inappropriate sampling strategy may lead to excessive resource utilization when the same purpose could have been served by a smaller sample with less resource and time consumption. On the other hand, if sampling is done poorly the results from the study may yield misleading inferences and may not have validity for generalization. Sampling could be probabilistic where sampling units – mostly individuals but can be households, villages etc – have known probability of getting included in the study; or it could be non-probabilistic where such is not the case. Probabilistic sampling is usually the preferred sampling method for majority of the epidemiological situations and studies. However, it should be remembered that non-probability sampling could be of immense value in certain situations [1].

When the requirement pertains to monitoring and evaluation, the importance of getting relevant data in quick time cannot be overemphasized. Study designs employing larger sample sizes but giving relatively robust results may not be appropriate for such requirements except for midterm or end term evaluation of programs, schemes, projects etc. Non-probability sampling strategies are not preferred for lack of their representativeness.

To overcome the difficulty of time and resource constrain while commanding reasonable robustness and representativeness of study findings, certain rapid survey methods have been developed and are in wide use. More known and frequently used such method is 30 by 7 cluster sampling method for assessing vaccination coverage in community settings [2]. Other rapid survey methods include ‘Lot Quality Assurance Sampling (LQAS) method and 100 Household survey method [3]. LQAS has attracted attention of program managers for the purpose of monitoring and evaluation of large campaigns/ programs including national programs [4], it is imperative that LQAS as a survey method be given its due academic importance. In this paper we discuss LQAS, with emphasis on methods and common pitfalls while interpreting and drawing conclusions from data collected through LQAS. This paper is primarily addressed to the post graduates in the departments of community medicine, community and family medicine, preventive and social medicine, social and preventive medicine, those pursuing master’s in public health, program managers, monitoring and evaluation professionals working in health care sector and the faculties of these departments and schools of public health across the country and thereafter.

### LQAS and its Methodology

Lot Quality Assurance Sampling is sometimes also called as ‘acceptance sampling’, as we generally select administrative unit/s which is/are accepted or rejected based on their performance in LQAS against a predetermined acceptable cut off value [5]. The administrative units are selected in such a way that they provide meaningful result to the planning unit for planning and action. However, all lots within an administrative unit need to be included for sampling once they are defined and identified as is the case with the stratified sampling strategy [6,7].

From the epidemiological point of view, the null hypothesis (Ho) formulated in LQAS for health care related outcomes is that an administrative unit - which could be a village, subhealth centre, Primary Health Centre (PHC), Community Health Centre (CHC) or District as a whole - has coverage of a health service or related activity of interest, less than a specified minimum level. The alternative hypothesis (Ha) states that the administrative unit has high coverage of a health service or related activity of interest, than a specified level. This clearly shows that the test applied in LQAS for statistical testing purpose is always one sided [6,7].

LQAS allows the researcher to draw inferences both at the sampling unit level and to extrapolate the results after combining the sampling units for the larger sampling frame – a desirable quality in programmatic conditions - which is not the case with other rapid assessment techniques [7,8,9].

Sampling Frame: Sampling frame for LQAS consists of all discrete smallest geographical units (lots) of interest under a defined larger administrative unit of interest. For example, in a state the districts may be counted as the smallest administrative unit. Thus, all districts will feature in the sampling frame. On other hand, if District is the larger administrative unit of interest, all the villages, or Sub-health centers, or the Primary Health Centres or the sub-districts may form the sampling frame depending upon the purpose of the enquiry and the level/s at which it need to be answered.

Lots: The geographical area is segregated into the sampling units or lots - which are the smallest administrative units - having implications for use of the data generated through LQAS towards improvement of services. In Indian context, they may be the ‘Districts’ or ‘Blocks’ or other relevant administrative unit like CHC, PHC, health sub-centre depending upon the programmatic requirements.

Sample Size: Selection of appropriate sample size for LQAS depends upon multiple factors as is the case with any other epidemiological study. However, for LQAS the investigator needs to predefine a minimum level of acceptable coverage and an upper level of desirable or targeted coverage (in case of negative outcome it can be reversed) against which the lot will be assessed together with the maximum acceptable ‘alpha (α)’ and ‘beta (β)’ errors. From these the total sample size of the LQAS is calculated in a two step process: In the first step, a sample size (n) which yields a test with stated ‘α’ and ‘β’ errors for the particular null and alternative hypothesis specified is chosen - this can also be called as the necessary minimum number ‘n’ required. This can be calculated using the formula:

‘n’= [Z_1-__α_{P_o_(1-P_o_)}^0.5^+ Z_1-β_ {P_a_(1-P_a_)}^0.5^]^2^/(P_a_-P_o_)^2^

Where Po is the lower acceptable coverage to accept a lot and Pa is the upper cut-off chosen as described above.

In the subsequent step, the value of d* (decision cut-off’number/value) for the necessary ‘n’ is determined. This number/value is determined using a binomial distribution. In brief, binomial distribution is applicable when there are only two outcomes possible, one of which is labeled as ‘success’ and other as ‘failure’. For a given number of trials, the probability of success or failure can be calculated. For example, coin toss can be considered a trial, where only two outcomes – heads and tail – are possible. If we label ‘heads’ as success, using binomial equations we can accurately calculate probability of ‘x’ success in ‘y’ trials. In LQAS, each individual sample is treated like a trial. Minimum number of success required for a given probability forms the basis of deciding d*. Without going into the exact binomial formulas and its steps, the decision cutoff value, d* for LQAS purpose can be approximately calculated using the formula:

d* = [(nP_o_) - Z_1-__α_{nP_o_(1-P_o_)}^0.5^]

Thus, we get two values while determining sample size in LQAS, first ‘n’ specifies the number of samples required in a lot and d* specifies the cutoff value/ number to determine whether a lot should be accepted or rejected [10].

However, as is the case with all epidemiological studies the total calculated sample size should be a fine balance between the accuracy of result required and the resources available while keeping purpose of the study firmly in mind. Tables for sample size requirements have been included in this article, so that researchers need not perform calculations, if they wish to avoid them (Table-1, 2).

### Sampling Strategy

Quality of LQAS depends upon randomness of the samples included in the study. However, actual sampling strategy in real world depend up on the type of data available about the population/ sampling units within a lot.

1. If list of all eligible individuals is available, then the best method will be the Simple Random Sampling (SRS). From the list of all eligible individuals the required individuals can be randomly selected (using random number table or any other appropriate random number generator). This is usually a very cumbersome, protracted exercise in most of the programmatic situations and not usually applicable.
2. If the list of households is available, then the preferred method will be selection of the households by simple or systematic random sampling. From the selected households, eligible individuals can be selected. Again in programmatic settings this may not be available and may need a separate exercise. If the selected household contains only one eligible individual then that individual gets included in the study. However, If there are multiple eligible individuals within a household, then any predefined random selection method should be used to select one individual. If no eligible individual is available, then the immediate next household (or any other appropriate replacement rule) can be identified and eligible individual is included.
3. If household list is also not available, then the lot (unit) can be sub-divided. The ‘grid method’ - where a grid with 100 compartments of equal size is used - can be used. The grid is placed over the map of the selected geographical area. The grid compartments are numbered systematically, followed by selection of required number of grid compartments using any standard random selection method. In the next step households can be selected randomly. Selection of households in the grid method can be done by using simple or systematic random sampling. In the selected households, eligible individual can be selected next as described above.

### Illustrative Example

Suppose we want to assess the coverage of antenatal care services in a District. The indicator chosen for the same is ‘At least one ANC visit during the pregnancy’. The study population for this can be all women who delivered in say last 6 months (or 1 year) of beginning of the study data collection. The smallest reporting and implementation unit in this case is taken to be PHC. So, samples must be taken considering geographical area under each of the PHC as sampling units. In a PHC, the list of delivered women is readily available with the medical officer in-charge. From this required number of women that need to be included in the study can be randomly sampled and surveyed. Selecting the lower and upper cutoffs can be tricky in such cases. Ideally the coverage should be 100%, but for the purpose of study the researcher needs to select lower and upper cut off values. Reported state level coverage can be used as a benchmark to take it as the minimum acceptable/lower cut off value. Upper cut off value can be the stated targets to be achieved by a programme, however, on most occasions its a judgement call for the purpose of feasibility of sample size arrived at when using different such cut offs. If the upper cut off is taken as 80% and the lowest acceptable cut-off is 70%, then the decision cut off number for the individual PHC to count as success will be 116 and the total sample size needed will be 156. The maximum number of eligible women available within a PHC area at any given time will only be around 330, given the current national birth rates and the population norm for a typical PHC area in India. Reaching required 156 women who have delivered in last 6 months in each of the PHC areas will be very laborious and resource consuming exercise – defeating the very purpose of rapid assessment. Thus, need of readjustment of cut off values in the study. If the upper cut off value is taken as 90% and the lower cutoff to be 65% the sample size required dramatically reduces to only 20 individuals with cut off decision value of 15 – very much possible in a short period of time and with relatively meagre consumption of resources.

### LQAS Variants

Traditional LQAS has upper and lower cut-off values along with predefined allowable alpha and beta errors to reach a sample size and the number of success required to reach a decision. However, there are few modifications of this method used/ suggested in the literature. One of such variants is ‘Multiple Category-LQAS’, where the traditional LQAS sample is classified using multiple decision rules. That means, rather than classifying vaccination coverage as being 50% or above in a population or not, it could be classified into as being low (say below 50%), high (say 80% or above) or moderate (in between). Thus, if we have three categories of decision making, we can calculate a sample size ‘n’ and the corresponding decision rules d1, d2 rather than only one decision rule d* [11]. A slight modification also includes double sampling plan for two different decision cutoff numbers d1, and d2 – it is essentially double stage sampling to conserve resources if the results are extreme in first stage sampling itself. If in the first stage, the number of individuals with desirable intervention is more than d1 but less then d2, only then second stage sampling is done [12]. Another variant of traditional LQAS is ‘Curtailed’ and ‘Semi-curtailed’ variants of LQAS, whereby if the required success are met before the complete sample has been covered, no more samples need to be covered (Semi-curtailed). On the other hand, if before completion of complete sample coverage, such number of failures have been encountered that required number of success for decision making are not possible from the remaining sample, further sampling can be stopped (Curtailed). Curtailed and Semi-curtailed variants help in maximum saving of time and resources while not compromising on the LQAS decision-making ability [11]. Another variant of traditional LQAS is Large Country – LQAS (LC-LQAS) where rather than selecting all units within the sampling frame for random selection of individuals, cluster approach can be used to get estimates for both local as well as larger national or sub-national regions. LC- LQAS is largely useful in situations where capacity of a country is limited and inclusion of all units may not be possible simultaneously [9].

### Drawing inferences from LQAS

LQAS results should only be interpreted qualitatively at the lot level i.e. either the population has adequate or inadequate coverage for the chosen cut-off. Results for larger geographical region/ State/ Country can be determined quantitatively. Traditionally, in LQAS null hypothesis is formulated towards the conservative side that is, the null hypothesis assumes less coverage for beneficial health interventions like vaccination (and opposite for harmful events like drug adverse effects or AEFI). LQAS is designed in such a way that strong evidence is required for rejecting the null hypothesis. For clarity, if we take an example of vaccination coverage of a lot (say one PHC) where n = 19 and d* 13 are determined with lower and upper vaccination coverage cutoff chosen as 50% and 80% respectively, then to reject null hypothesis (coverage less than 50%) we need 13/19 = 68.4% randomly selected individuals who are vaccinated. Even when the null hypothesis is rejected (i.e. the decision cut off value is met) the prudent inference is that the lot (PHC) has vaccination coverage of at least 50%. One common error is made in thinking that if the decision cut off value is met then the null hypothesis stands rejected and the coverage can be taken as 80%, which is not correct. Thus, strong evidence is used to make conservative inferences, to protect against any errors due to small sample sizes typically used in LQAS [13].

Another pitfall researcher needs to be careful of while drawing inferences is about the way the null hypothesis is formulated. If null hypothesis is turned upside down i.e. it assumes that the coverage is high (say 80% and lower cut off used is 50%) and it gets rejected using n=19 and d*=13 decision criteria; then again, the chances of wrong inference are high as 68.4% vaccinated individuals out of 19 random individuals are leading the researcher to conclude that the coverage is high (80%). Thus such framing of null hypothesis may cause harm to the population as resources or special activities may be withdrawn once an inference (even if erroneous) is made that the coverage of services is high [13].

We therefore recommend, as has been recommended elsewhere also, that a conservative null hypothesis should be used wherever LQAS is used in programmatic settings so that interest of public at large are protected even if it means excess service delivery due to higher probability of making type II error.

### LQAS uses at present

LQAS has been widely used for assessment of coverage in special campaigns. In India it is presently being used in the National Vector Borne Diseases Control Program (NVBDCP), where decision making is based on 19-13 criteria [14]. Nineteen random individuals are sampled for the survey, if 13 success are met the coverage is considered to be satisfactory for the programmatic purposes [14]. Individual studies have been done in India where researchers have used LQAS to assess vaccination coverage and other health related activities [15,16,17].

Outside India, in Bangladesh LQAS is currently being used for assessing immunization coverage and in monitoring the elimination of neonatal tetanus [18]. Similar to India, in Mozambique LQAS is in use to assess malaria programme of that country [19]. Outside programmes, LQAS has also been used in individual studies to evaluate health worker performance, coverage of maternal, new born and child health services etc [20,21]. In a study comparing LQAS to surveillance, it was not found inferior to surveillance [22].

## Conclusion and way forward:

In our view, if used appropriately, LQAS is a very effective tool at the disposal of researchers working within the health sector - more so for those involved in the monitoring and evaluation activities. Twin benefits of robust results when conservative hypothesis is used, along with quick availability of results and straightforward decision making criteria make it especially attractive for these purposes. However, in our view, it is being underutilized at present due to a variety of reasons including lack of familiarity among researchers with this method, limited pedagogy discussions during academic/training period and slightly different methods of drawing and interpretation of inferences from LQAS studies, resulting in professionals avoiding this method for their study purposes. This paper hopefully will be a step towards correcting some of these issues.

## Figures and Tables

**Table 1:** Required sample size and decision cutoff value for given upper and lower percent coverage for α=5, β= 90 (Adapted from Lemeshow S et al. Adequacy of Sample Size in Health Studies. World Health Organization. 1990) [10]

%	50		55		60		65		70		75		80		85		90		95	
5	**6**	**0**	**5**	**0**	**-**	**-**	**-**	**-**	**-**	**-**	**-**	**-**	**-**	**-**	**-**	**-**	**-**	**-**	**-**	**-**
10	**10**	**2**	**8**	**2**	**6**	**1**	**5**	**1**	**-**	**-**	**-**	**-**	**-**	**-**	**-**	**-**	**-**	**-**	**-**	**-**
15	**14**	**3**	**11**	**3**	**8**	**2**	**7**	**2**	**5**	**1**	**-**	**-**	**-**	**-**	**-**	**-**	**-**	**-**	**-**	**-**
20	**20**	**6**	**15**	**5**	**11**	**3**	**9**	**3**	**7**	**2**	**5**	**2**	**-**	**-**	**-**	**-**	**-**	**-**	**-**	**-**
25	**31**	**10**	**21**	**7**	**16**	**6**	**12**	**5**	**9**	**4**	**7**	**3**	**5**	**2**	**-**	**-**	**-**	**-**	**-**	**-**
30	**50**	**19**	**32**	**12**	**22**	**9**	**16**	**7**	**12**	**5**	**9**	**4**	**7**	**3**	**5**	**2**	**-**	**-**	**-**	**-**
35	**92**	**38**	**52**	**22**	**33**	**15**	**22**	**10**	**16**	**8**	**11**	**5**	**8**	**4**	**6**	**8**	**5**	**3**	**-**	**-**
40	**211**	**93**	**93**	**43**	**52**	**25**	**32**	**16**	**22**	**11**	**15**	**8**	**11**	**6**	**8**	**5**	**6**	**4**	**-**	**-**
45	**853**	**402**	**212**	**104**	**93**	**28**	**51**	**27**	**31**	**17**	**21**	**12**	**14**	**8**	**10**	**6**	**7**	**4**	**-**	**-**
50			**852**	**444**	**210**	**114**	**91**	**51**	**49**	**29**	**30**	**18**	**19**	**12**	**13**	**8**	**9**	**6**	**5**	**3**
55					**834**	**477**	**203**	**120**	**87**	**53**	**46**	**29**	**27**	**18**	**17**	**12**	**11**	**8**	**7**	**5**
60							**798**	**496**	**191**	**123**	**80**	**53**	**42**	**29**	**24**	**17**	**14**	**10**	**8**	**6**
65									**746**	**501**	**176**	**122**	**72**	**52**	**36**	**27**	**20**	**15**	**11**	**9**
70											**676**	**488**	**156**	**116**	**62**	**48**	**30**	**24**	**15**	**12**
75													**589**	**455**	**131**	**104**	**49**	**40**	**21**	**18**
80															**484**	**398**	**102**	**86**	**34**	**30**
85																	**362**	**316**	**67**	**60**

**Table 2:** Required sample size and decision cutoff value for given upper and lower percent coverage for α =10, β =90 (Adapted from Lemeshow S, et al. Adequacy of Sample Size in Health Studies. World Health Organization. 1990) [10]

%	50		55		60		65		70		75		80		85		90		95	
**5**	**5**	**1**																		
**10**	**7**	**1**	**6**	**1**	**5**	**1**														
**15**	**10**	**2**	**8**	**2**	**6**	**2**	**5**	**1**												
**20**	**15**	**5**	**11**	**3**	**9**	**3**	**7**	**2**	**5**	**2**										
**25**	**23**	**8**	**16**	**6**	**12**	**5**	**9**	**4**	**7**	**3**	**5**	**2**								
**30**	**38**	**15**	**25**	**10**	**17**	**7**	**12**	**5**	**9**	**4**	**7**	**3**	**5**	**2**						
**35**	**70**	**29**	**39**	**17**	**25**	**11**	**25**	**13**	**12**	**6**	**9**	**5**	**7**	**4**	**5**	**3**				
**40**	**161**	**72**	**72**	**34**	**40**	**20**	**39**	**21**	**17**	**9**	**12**	**7**	**9**	**5**	**6**	**3**	**5**	**3**		
**45**	**654**	**310**	**163**	**81**	**72**	**37**	**70**	**40**	**25**	**14**	**16**	**9**	**11**	**7**	**8**	**5**	**6**	**4**		
**50**			**654**	**343**	**161**	**88**	**156**	**93**	**38**	**22**	**23**	**14**	**15**	**10**	**9**	**7**	**7**	**5**	**5**	**4**
**55**					**641**	**368**	**615**	**384**	**67**	**42**	**36**	**23**	**22**	**15**	**14**	**10**	**9**	**6**	**6**	**5**
**60**									**148**	**96**	**63**	**42**	**33**	**23**	**29**	**22**	**12**	**9**	**7**	**5**
**65**									**575**	**388**	**137**	**96**	**57**	**41**	**49**	**38**	**16**	**12**	**9**	**7**
**70**											**522**	**378**	**121**	**91**	**103**	**82**	**24**	**19**	**13**	**11**
**75**													**456**	**353**	**377**	**311**	**40**	**33**	**18**	**15**
**80**																	**81**	**69**	**28**	**25**
**85**																	**284**	**249**	**55**	**50**

## References

[1] Acharya AS Prakash A Saxena P Nigam A. Sampling: Why and How of it? Indian Journal of Medical Specialities. 2013;4(2):330-3. 10.7713/ijms.2013.0032

[2] EPI coverage Survey, World Health Organization, [online] 2008 [cited 2018 Dec 25] Available from: https://www.who.int/immunization/documents/MLM_module7.pdf

[3] (2003). Immunization Essentials, USAID, [online].

[4] Robertson SE Valadez JJ Global review of health care surveys using lot quality assurance sampling (LQAS), 1984-2004. Soc Sci Med. 2006 Sep;63(6):1648-60. Epub 2006 Jun 9. Review. 10.1016/j.socscimed.2006.04.011 PubMed PMID: . 16764978

[5] Hund L New tools for evaluating LQAS survey designs. Emerg Themes Epidemiol. 2014 Feb 15;11(1):2. doi: 10.1186/1742-7622-11-2. 10.1186/1742-7622-11-2 PMid: PMCid: 24528928 PMC3931287

[6] Measure Evaluation: Report of a Technical Meeting on the Use of Lot Quality Assurance Sampling (LQAS) in Polio Eradication Programs. 1998, Carolina Population Center, University of North Carolina, [online] 1998 [cited 2018 Dec 25] Available from: https://www.measureevaluation.org/resources/publications/wp-98-10/at_download/document

[7] Robertson SE Anker M Roisin AJ Macklai N Engstrom K LaForce FM The lot quality technique: a global review of applications in the assessment of health services and disease surveillance. World Health Stat Q. 1997;50(3-4):199-209.Review. PubMed PMID: . 9477550

[8] Lanata CF Black RE Lot quality assurance sampling techniques in health surveys in developing countries: advantages and current constraints. World Health Stat Q. 1991;44(3):133-9. 10.1016/0952-3278(91)90197-D PubMed PMID: . 1949880

[9] Hedt BL Olives C Pagano M Valadez JJ Large Country-Lot Quality Assurance Sampling: A New Method for Rapid Monitoring and Evaluation of Health, Nutrition and Population Programs at Sub-National Levels. HNP Discussion paper; The World Bank, [online] 2008 [cited 2018 Dec 25] Available from: http://documents.worldbank.org/curated/en/259011468333269796/pdf/834240WP0Large0Box0382086B00PUBLIC0.pdf

[10] Lemeshow S Hosmer DW Jr Klar J Lwanga SK Adequacy of Sample Size in Health Studies. World Health Organization 1990.

[11] Olives C Valadez JJ Brooker SJ Pagano M. Multiple Category-Lot Quality Assurance Sampling: A New Classification System with Application to Schistosomiasis Control. PLoS Negl Trop Dis 6(9): e1806. 10.1371/journal.pntd.0001806 PMid: PMCid: 22970333 PMC3435238

[12] Lemeshow S Taber S. Lot quality assurance sampling: single- and double-sampling plans. World Health Stat Q. 1991;44(3):115-32. PubMed PMID:. 1949879

[13] Rhoda DA Fernandez SA Fitch DJ Lemeshow S. LQAS: User Beware. Int J Epidemiol 2010;39:60-8. 10.1093/ije/dyn366 PMid: 20139433

[14] Operational Manual for Malaria Elimination in India,. 2016, National Vector Borne Disease Control Programme, Govt. of India, [online] 2016 [cited 2019 Jan 10] Available from: http://www.nvbdcp.gov.in/index1.php?lang=1&level=1&sublinkid=5830&lid=3686

[15] Punith K Lalitha K Suman G Pradeep BS Kumar KJ Evaluation of Primary Immunization Coverage of Infants Under Universal Immunization Programme in an Urban Area of Bangalore City Using Cluster Sampling and Lot Quality Assurance Sampling Techniques. Indian J Community Med. 2008; 33(3): 151-5. 10.4103/0970-0218.42049 PMid: PMCid: 19876474 PMC2763683

[16] Datta A Baidya S Datta S Mog C Das S. A Study to Find Out the Full Immunization Coverage of 12 to 23-month old Children and Areas of Under-Performance using LQAS Technique in a Rural Area of Tripura. J Clin Diagn Res. 2017; 11(2): LC01-LC04. 10.7860/JCDR/2017/23919.9428 PMid: PMCid: 28384892 PMC5376832

[17] Piot B Mukherjee A Navin D Krishnan N Bhardwaj A Sharma V Lot quality assurance sampling for monitoring coverage and quality of a targeted condom social marketing programme in traditional and non-traditional outlets in India. Sex Transm Infect. 2010; 86(Suppl_1): i56-i61, PubMed, PMID: 20167732 10.1136/sti.2009.038356 PMid: PMCid: 20167732 PMC3252603

[18] Tawfik Y Hoque S Siddiqi M. Using lot quality assurance sampling to improve immunization coverage in Bangladesh. Bull World Health Organ. 2001;79(6):501-5. PubMed PMID: ; PubMed Central PMCid: . 11436470 PMC2566438

[19] Biedron C Pagano M Hedt BL Killian A Ratcliffe A Mabunda S An assessment of Lot Quality Assurance Sampling to evaluate malaria outcome indicators: extending malaria indicator surveys Int J Epidemiol. 2010;39(1):72-79. 10.1093/ije/dyp363 PMid: PMCid: 20139435 PMC2912491

[20] Mwanza M Zulu J Topp SM Musonda P Mutale W Chilengi R. Use of Lot quality assurance sampling surveys to evaluate community health worker performance in rural Zambia: a case of Luangwa district. BMC Health Serv Res. 2017:17(1):279. 10.1186/s12913-017-2229-9 PMid: PMCid: 28416009 PMC5393033

[21] Abegunde D Orobaton N Shoretire K Ibrahim M Mohammed Z Abdulazeez J Monitoring maternal, newborn, and child health interventions using lot quality assurance sampling in Sokoto State of northern Nigeria. Glob Health Action. 2015;8:27526. 10.3402/gha.v8.27526 PMid: PMCid: 26455491 PMC4600711

[22] Bhuiya A Hanifi SMA Roy N Streatfield PK Performance of the Lot Quality Assurance Sampling Method Compared to Surveillance for Identifying Inadequately-performing Areas in Matlab, Bangladesh. J Health Popul Nutr. 2007 Mar; 25(1): 37-46. PubMed, PMID: . 17615902 PMC3013262

